# An investigation of improving validity in upper limb measurements for people with tetraplegia using construct specification equations

**DOI:** 10.1038/s41598-025-98626-4

**Published:** 2025-08-04

**Authors:** Johanna Wangdell, Leslie Pendrill, Jennifer A. Dunn, Bridget Hill, Jeanette Melin

**Affiliations:** 1 Centre for Advanced Reconstruction of Extremities, Sahlgrenska University Hospital/Mölndal, Mölndal, Sweden; 2 Department of Hand Surgery, Institute of Clinical Sciences, University of Gothenburg, Gothenburg, Sweden; 3 Division of Safety and Transport, Measurement Science and Technology Unit, RISE Research Institutes of Sweden, Gothenburg, Sweden; 4 Department of Orthopaedic Surgery and Musculoskeletal Medicine, University of Otago, Christchurch, New Zealand; 5 Department of Plastic and Reconstructive Surgery, Austin Health, Melbourne, Australia; 6 Epworth Health Care, Melbourne, VIC Australia; 7 Department of Leadership, Demand and Control, Swedish Defence University, Karlstad, Sweden

**Keywords:** Task difficulty, Person ability, Construct specification equation, Validation, Rasch analysis, TUAQ, Medical research, Neurology

## Abstract

The aim of this paper is two-fold: to investigate development of a Construct Specification Equation (CSE) for UL task difficulty, , and a CSE for person UL ability, , in support of the validity of these two constructs. Measurements of UL task difficulty, , and person UL ability, were derived from applying the Rasch model on the Tetraplegia Upper Limb Activity Questionnaire (TUAQ). The formulations of CSEs as explanations of the two constructs were done using Principal Component Regression (PCR). The CSE for UL task difficulty, , was to a large degree explained by the number of joints involved and the CSE for person UL ability, , was dominated by grasp-related variables. Pearson coefficients of 0.94 and 0.73 were obtained between UL task difficulty and UL person ability from the CSE, respectively, when correlated with each empirical measure. The present work has both explored and extended the methodology for using more qualitative explanatory variables. Specifically, for UL measurements for people with tetraplegia a good CSE for task difficulty, , supports the validity of TUAQ when measuring person UL ability. Additionally, the CSE formulated for person ability, , can be used both for validation purposes as well as a clinical tool.

**Supplementary Information:**

The online version contains supplementary material available at 10.1038/s41598-025-98626-4.

## Introduction

Sustaining a cervical spinal cord injury leads to tetraplegia. This results in paralysis in the legs and to a various extent in the arms and hands. Most daily activities rely on the use of the upper limb (UL) and even more so if the legs are paralysed. The need to optimise and restore arm- and hand function within rehabilitation is therefore fundamental. Improvements in hand function has been rated as the most important function to regain by persons living with tetraplegia^1,2^.

In the field of reconstructive UL surgery in tetraplegia the need for standardised and well-targeted outcome measures has been highlighted^3,4^. There is a crucial need for rehabilitation clinicians and researchers to have valid and reliable measurements to diagnose, treat and rehabilitate consistently throughout the health care system^5–7^. In particular, the need for reliable patient centred outcome measures targeting the specific daily activities relevant when living with a tetraplegia has been identified^4^. To address this need, the Tetraplegia Upper Limb Activity Questionnaire (TUAQ) was developed^8^. The TUAQ has been analyzed according to the Rasch model and demonstrates sound initial psychometric properties^8^. It is recommended for use in both clinical and research settings to measure patients’ UL ability^8,9^.

A person who has more of a latent trait – here UL ability – is more likely to score higher on a difficult item in TUAQ than a person who has lower UL ability. Separate estimates of each task difficulty, δ, and person ability, , can be made with the Rasch model as it provides estimates of person ability, , on a linear scale rather than on the non-linear scale of the ordinal raw scores. At the same time, the Rasch model also provides estimates of task difficulty, δ, on a linear scale (in fact, conjoint with the ability scale) and locates the items along a continuum, from easier tasks to more difficult tasks. For polytomous items, task difficulty, δ, is determined as the average of the item thresholds (i.e., an item threshold is the point where the probability of selecting a higher response category exceeds that of a lower category). If the items’ locations correspond to a clinical expected continuum, the hierarchy of item order provides evidence towards the construct validity^10^. The TUAQ item hierarchy study indicated that easier items were less complex (e.g., with fewer joints involved and less coordination required between the left and right arms) compared with the more difficult items^8^. The conjoint scale for person ability and task difficulty means that an item hierarchy can inform rehabilitation clinicians and researchers on where the patient is located on the continuum, where he or she comes from and what tasks are next^11^. As such, it can be used in planning rehabilitation to make sure the patient’s rehabilitation path gradually evolves according to the patient’s ability.

While a Rasch analysis provides the necessary transformation of raw data, it does not explain *why*a person is located where he or she is or why a task is easier or more difficult. Previously, the role in establishing validity and improving test accuracy of construct specification equations (CSEs) as specific, causal, and rigorously mathematical explanations of person ability and task difficulty, respectively, has been stressed^12^. Citing Stenner and colleagues^13^: *The primary characteristic setting the concept of the specification equation apart from other approaches to psychological and social measurement is its decidedly mechanismic (causal) approach*. As in any modelling, such equations can both describe the actual status based on observations as well as enable predictions of future results to be expected. Such predictions can apply both to each principal construct (task difficulty, δ, and person ability, ) as well as contributions to these overall constructs from the various explanatory variables. Causality as opposed to mere correlation needs of course to be verified in each particular case, which is a topic of much current debate^14^.

Thus, such explanatory models could advance the construct validity of TUAQ. Despite being introduced as early as the 1980 s^15,16^, the application and development of CSEs have been relatively slow in health care. Over the years, in the little work that has been done on CSEs in health care, the emphasis has alternated between focusing on task difficulty and on person ability^15^. More recently we have argued for starting with developing CSEs for task difficulty, , as tasks are, in general, conceptually simpler and more robust than person ability^17^. However, with well-designed measurement systems, a CSE for person ability can also be used in many cases both for validation purposes and as a clinical tool to predict outcomes (see further discussion in Sect."Discussion")^12^.

To date, most of the work on CSE for task difficulty, , has included quantitative explanatory variables (such as entropy, sequence length, word frequency)^12,15–18^. In some cases, qualitative variables have been included as explanatory factors in CSE, such as certain material properties (such as rheological)^19^. Other related work has started exploring the possibilities of including more qualitative variables when explaining task difficulty, ^20–23^. For instance, Adroher and Tennant^20^ proposed that clinical judgments can be used to explain why certain items are easier or more difficult to advance the construct validity. In their study, they recruited professionals to rate all items of the Evaluation of Daily Activity Questionnaire (EDAQ) according to seven explanatory variables: overall physical demand, bilateral hand involvement, fine hand use, physical endurance, overall cognitive demand, sequence complexity, and concentration. The professionals’ ratings were then used to predict task difficulty and compared with empirical data estimates of EDAQ task difficulties. A similar approach has been adopted by Seamon and colleagues^21^, where gross motor development could be explained by the degree of body position, movement and support. Specifically, they present a framework for both understanding and measurement of gross motor development, and in turn propose that it enables creating a universal scale and unit. Such advances should be applicable as well to the TUAQ in focus in the present study, thereby further advancing the understanding of the validity of the construct UL task difficulty.

To conclude this Introduction, when ensuring validity in measurements of person abilities and task difficulty, such as in UL measurements, CSEs are useful as specific, causal, and rigorously mathematical construct explanations. Given the initial sound psychometric properties of TUAQ and its clinically relevant item hierarchy^8^ and the recent emphasis about CSE as one of the most advanced construct theories to enhance construct validity^18,24^, the aim of this paper is two-fold: first, we will investigate the specific components required for explaining UL task difficulty in the TUAQ in support of construct validity. We will do this by application of a CSE methodology using qualitative explanatory variables derived from professionals’ ratings for UL task difficulty, . Secondly, by developing another CSE for person UL ability we will ascertain relationships between sets of objective measures and person UL ability, .

## Materials and methods

### Participants

The participants in this study originate from earlier work^8^ and were recruited from two specialised units for reconstructive upper limb surgery for persons with tetraplegia, at Sahlgrenska University Hospital, Gothenburg, Sweden and in Burwood Hospital, Christchurch, New Zealand. Inclusion criteria in the main study was subjects had sustained a cervical spinal cord injury and undergone reconstructive surgery with the aim to improve UL function and grip ability. Exclusion criteria were other injuries or limitations in their upper limb. Informed written consent was obtained from all participants and the study were approved by ethics committee of Goteborg(D-nr:099 − 16) and University of Otago Ethics Committee (Health) ref 18/055) and research was performed in accordance with the Declaration of Helsinki.

To be included in this study, for developing the CSE for person ability, the subject should have a measure of all significant explanatory variables. This revealed a subsample of 67 patients. The subsample person characteristics are presented in Table 1. Details about time post injury, type and number of surgeries together with outcome measures targeting grip ability and grip strength are further presented in Sect."Tetraplegia upper limb activities questionnaire (TUAQ)")", which were used as explanatory variables in the development of a CSE for person ability.

**Table 1:** Person and injury characteristics for CSE for person UL ability.

	*n* = 67
Gender *	
Men (n, %)	37 (56%)
Women (n, %)	29 (44%)
Age (mean, SD)	39.8 (17.7)
ISNCSCI Motor level *	
C4	2 (3%)
C5	10 (15%)
C6	18 (27%)
C7	31 (46%)
C8	3 (4%)
AIS*	
A	40 (60%)
B	14 (21%)
C	4 (6%)
D	6 (9%)
Limiting spasticity in UL *	
Yes	47 (70%)
No	14 (21%)
Time post injury (TPI) [years] (mean, SD)	4.7 (4.3)
Timepoint; pre or post-surgery (TPPS)	
Pre surgery (n, %)	27 (41%)
3 months (n, %)	8 (12%)
6 months (n, %)	9 (13%)
12 months (n, %)	23 (34%)
Previous UL surgeries (pUL_surg) (n, %)	
0 (n, %)	47 (70%)
1 (n, %)	13 (19%)
2 (n, %)	7 (11%)
Grip strength [kg] (mean, SD)	2.82 (3.46)
Pinch strength [kg] (mean, SD)	1.1 (1.26)
GRT [count] (mean, SD)	80 (60)

* One person missing for gender, three persons missing for ISNSCI and AIS, six persons for limiting spasticity.

AIS = American Spinal Injury Association Impairment Scale; GRT = Grasp and release test; ISNCSCI = International Standards for Neurological Classification of SCI; pUL_surg = Previous UL surgeries; TPI = Time post injury; TPPS = Timepoint of assessment pre or post-surgery.

### Construct specification equations (CSEs)

As explained in the Introduction, CSEs play a key role in establishing validity and improving test accuracy in any kind of measurement of latent traits. The formulation of CSE for the construct *Z* – which could be a prediction of either of the two conjoint attributes (UL task difficulty, , or person UL ability, , as the dependent variable) – is often formulated as a linear combination of a set, *k*, of explanatory (The extent to which a certain variable is ”explanatory” will be ultimately determined by a thorough investigation of causality (as opposed to mere correlation).) (independent) variables, *X*:

1 $$\widehat{Z}=\sum_{k}{\beta}_{k}\cdot{x}_{k}$$

A comprehensive description on the CSE methodology can be found elsewhere, but in short, it comprises of three steps of a principal component regression (PCR)^12,25^.

i. A principal component analysis amongst the set of explanatory variables, *X*_*k*_;
ii. A linear regression of the empirical task difficulty ${\widehat{Z}}_{j}=$*δ*_*j*_ or person ability ${\widehat{Z}}_{i}=$_*i*_ values against *X’=X • P* in terms of the principal components, *P*; and.
iii. A conversion back from the principal components to the explanatory variables, *X*_*k*_.

The formulation of a CSE can be viewed as an explorative work where a set of identified explanatory variables is evaluated iteratively with evaluations of model fit change when explanatory variables are varied. In turn, one seeks the best possible correlation (as measured in terms of the Pearson coefficient) between empirical task difficulty values, *δ*_*j*_, (or person ability _*i*_) and the predicted *Z*as well as the smallest measurement uncertainties in the β-coefficients^12^.

### Definitions, collections, and transformations of explanatory variables for UL task difficulty, 

The choice of the explanatory variables, X, which entered into the CSE (Eq. 1) is of course a key step. In most cases, one has chosen quantitative variables deemed most relevant when explaining the construct of interest – such as those characterizing, in memory tests, the level of difficulty of recalling a sequence, in terms of the number or degree of order of the objects (blocks, digits, words, etc.)^17^ – the longer and more unordered (more entropy) a sequence, the more difficulty it is to recall. In the present study, it is necessary to consider additionally *qualitative*explanatory variables. Building on the methodology proposed by Adroher & Tennant^20^ to advance CSE into fields without recourse to quantitative explanatory variables, a first requirement is to identify and define the explanatory variables that linearly increase or decrease ordinally along the continuum of hand function task difficulty. In this first step, it is important to define the demands required for the tasks themselves, not for the specific person/group of persons performing them. In the present study, this resulted in the identification of ten explanatory variables (Table 2). Key body functions and structures for UL activities were included, such as hand and arm strength, range of movement (ROM) and coordination. Based on Adroher & Tennant’s^20^ work, we also included sequence complexity as it was shown to be a promising variable for explaining activities of daily living. The selection and specification of potential variables at this stage were made by co-authors with extensive clinical and scientific experts in tetraplegia (JW, JD and BH) together with a literature review of previous studies of UL evaluation CSE. Furthermore, careful consideration was to not make the specifications too detailed to make sure that they could be read and easily understood by rehabilitation professionals in an online survey.

**Table 2:** Explanatory variables, instructions and response categories rated for all TUAQ items, J, respectively for the development of the CSE for UL task difficulty.

Explanatory variable	Instruction *	Response categories
Hand grip, ${{\delta}_{Hand\,grip}}_{j}$	Please rate the general demand of grip ability required to perform each of the tasks, *j*.	No grip ability demand required; Little grip ability demand required; Moderate grip ability demand required; Major grip ability demand required
Hand strength, ${{\delta}_{Hand\,strength}}_{j}$	Please rate the general demand of grip strength required to perform each of the tasks.	No grip strength demand required; Little grip strength demand required; Moderate grip strength demand required; Major grip strength demand required
Arm strength	Please rate the general demand of arm strength required to perform each of the tasks.	No arm strength demand required; Little arm strength demand required; Moderate arm strength demand required; Major arm strength demand required
Stabilization and positioning of the arm	Please rate the general demand of stabilization and positioning of the arm required to perform each of the tasks.	No stabilization and positioning of the arm required; Little stabilization and positioning of the arm required; Moderate stabilization and positioning of the arm required; Major stabilization and positioning of the arm required
Range of motion: Dominant hand	Please rate the general demand of range of motion for the dominant hand required to perform each of the tasks.	No range of motion for the dominant hand required; Little range of motion for the dominant hand required; Moderate range of motion for the dominant hand required; Major range of motion for the dominant hand required
Range of motion: Not dominant hand	Please rate the general demand of range of motion for the not dominant hand required to perform each of the tasks.	No range of motion for the not dominant hand required; Little range of motion for the not dominant hand required; Moderate range of motion for the not dominant hand required; Major range of motion for the not dominant hand required
Trunk control, ${{\delta}_{Trunk\,control}}_{j}$	Please rate the general demand of trunk control required to perform each of the tasks.	No trunk control required; Little trunk control required; Moderate trunk control required; Major trunk control required
Coordination	Please rate the general demand of coordination required to perform each of the tasks.	No coordination required; Little coordination required; Moderate coordination required; Major coordination required
Sequence complexity	Please rate the sequence complexity in each of the tasks.	No complexity; Little complexity; Moderate complexity; Major complexity
Number of joints involved, ${{\delta}_{Joints}}_{j}$	Please indicate *all* joints actively involved in each of the tasks.	Finger, Thumbs, Wrist, Pronation/Supination, Elbow, Shoulder

* For all explanatory variables, the following sentence was included, e.g.: Focus on the inherent demand for the grip strength of the task itself in the non-disabled population and not the performance of a specific patient.

Secondly, healthcare professionals (experienced occupational therapists and physiotherapists, *n* = 8) working in hand rehabilitation from our network were invited to score each of the identified explanatory variables (*n* = 10) for each item in the TUAQ. As the ratings from the healthcare professionals were ordinal data, a Rasch analysis with a partial credit model of the scored explanatory variables was needed to derive linear interval measures for each variable. Here, the focus of the Rasch analysis was on transforming the ordinal data for the explanatory variables, with less emphasis on full assessment of model fit. All except two reliability estimates of the explanatory variables were found to lie in the interval from 0.45 (*Arm strength*) to 0.68 (*Joints*); exceptionally, *Coordination* had a particularly low reliability of 0.24 and *Hand strength* had a particularly high reliability of 0.94. Descriptive statistics of the explanatory variables for UL task difficulty are provided in Supplement 1.

### Explanatory variables for person UL ability, 

For this study, characteristics specific for this patient group and typically collected as routine care were used as explanatory variables for person UL ability^4^, , when developing the possible CSE. Based on existing data we used six potential explanatory variables:

- Time post injury (TPI) after the spinal cord injury was used to capture the adjustment process required after the severe life changing event that a spinal cord injury is.
- Time pre- or post-surgery (TPPS) responses when the TUAQ was filled in, either the day before surgery or 3, 6, and 12 months after surgery.
- Number of previous UL surgeries (pUL_surg) (including bilateral surgeries).
- Grip strength was tested using Jamar Dynometer in standardized position and the maximum value from three attempts was reported^26,27^.
- Pinch strength was tested using Pinch Gauge in standardized position and the maximum value from three attempts was reported^26,27^.
- For grasp ability the Grasp and Release Test (GRT) was used. The GRT includes six items (peg, block, weight, fork, video and can) and the number of successful grasp and release attempts in 30 s for each item is recorded. The individual item scores are then combined to give a total score. The GRT has been psychometrically evaluated following implantation of a neuroprosthesis and tendon transfer surgery in a tetraplegic population^28^.

While TPI is related to the injury itself, TPPS and pUL_surg relate to the reconstructive surgery the study population underwent, and grip- and pinch strength as well as GRT are specifically related to UL ability.

### Tetraplegia upper limb activities questionnaire (TUAQ)

The TUAQ is a ten-item questionnaire targeting activities relying on arm and hand function and relevant for persons living with tetraplegia (Table 3). Responses to each question were rated on two 1–5 scales; experienced difficulties (1 = not able/5 = able extremely well) and experienced satisfaction with the performance (1 = not at all/5 = extremely satisfied). The ten items were selected from 714 goals identified by persons with tetraplegia prior to their reconstructive surgery. For the present work, only data for experienced difficulties were included.

**Table 3:** TUAQ items ordered from easiest to most difficult according to Wangdell et al.^8^. Responses to each question were rated on two 1–5 scales; experienced difficulties (1 = not able/5 = able extremely well) and experienced satisfaction with the performance (1 = not at all/5 = extremely satisfied).

	TUAQ items
1	Eating with a fork or spoon
2	Drinking from a bottle
3	Grasp and reposition book/tablet
4	Shaving/put on makeup
5	Writing with a pen (with your best hand)
6	Pick up items form a flat surface
7	Adjust upper half clothing pulling down back to waist level
8	Handle banknotes credit card in/out of wallet
9	Open previously opened jars
10	Cut food when eating (with your best hand)

The initial psychometric testing included 99 individuals with tetraplegia and showed fit to the Rasch model, including support for unidimensionality with no local dependency; no issues with item or person misfit; and minimal disordered thresholds^8^. Furthermore, the testing also demonstrated good test-retest reliability over two weeks among 47 individuals with tetraplegia and responsiveness for 33 individuals after UL surgery^9^. Nevertheless, to date, there have been no explanatory models attributed to the TUAQ.

## Results

### A construct specification equation (CSE) for UL task difficulty, 

Table 4 shows the extent of univariate correlation between the dependent variable, UL task difficulty, , retrieved from Wangdell et al. 2022^8^ and each of the ten potential explanatory variables presented in Sect."Explanatory variables for person UL ability, θ"(Table 3). Different combinations of explanatory variables were evaluated and by including the explanatory variables with the highest univariate correlations to UL task difficulty, ${\delta}_{j}$, for item *j*, in the PCR^25,29^ (as presented in Sect."Definitions, collections, and transformations of explanatory variables for UL task difficulty, δ"), the following CSE was derived:

2 $$\begin {aligned}{zR}_{TUAQ\,Task\,difficulty}&=1.2\left(6\right)+0.7\left(5\right)\cdot{{\delta}_{Hand\,grip}}_{j}+0.2\left(1\right)\cdot{{\delta}_{Hand\,strength}}_{j}\\&+0.4\left(3\right)\cdot{{\delta}_{Trunk\,control}}_{j}-0.5\left(1\right)\cdot{{\delta}_{Joints}}_{j}\end {aligned}$$

*Numbers in parentheses indicate measurement uncertainties (coverage factor, *k* = 2, according to JCGM 100:2008 Evaluation of measurement data — Guide to the expression of uncertainty in measurement, https://www.bipm.org/documents/20126/2071204/JCGM_100_2008_E.pdf/).

**Table 4:** Pearson correlation coefficients between task difficulty values from Wangdell et al.^8^ against all ten potential explanatory variables (Sect."Definitions, collections, and transformations of explanatory variables for UL task difficulty, δ") explored for the development of the CSE for UL task difficulty.

	Task difficulty Wangdell et al. 2022	Hand grip	Hand strength	Arm strength	Stabilization positioning arm	ROM Dominant	ROM Not Dominant	Trunk control	Coordination	Sequence complexity	Joints
Hand grip	**0.45**										
Hand strength	**0.48**	−0.03									
Arm strength	0.24	0.42	0.31								
Stabilization positioning arm	0.03	0.36	0.44	0.80							
ROM Dominant	−0.15	0.23	0.08	0.50	0.64						
ROM Not Dominant	0.22	0.41	0.24	0.74	0.60	0.71					
Trunk control	**0.51**	0.17	0.33	0.67	0.49	0.40	0.43				
Coordination	−0.14	−0.42	0.79	0.17	0.45	0.18	0.13	0.05			
Sequence complexity	−0.05	−0.48	0.63	0.13	0.32	0.33	0.24	0.14	0.81		
Joints	**−0.31**	0.01	0.30	0.60	0.79	0.75	0.66	0.36	0.56	0.47	

**Fig. 1:**
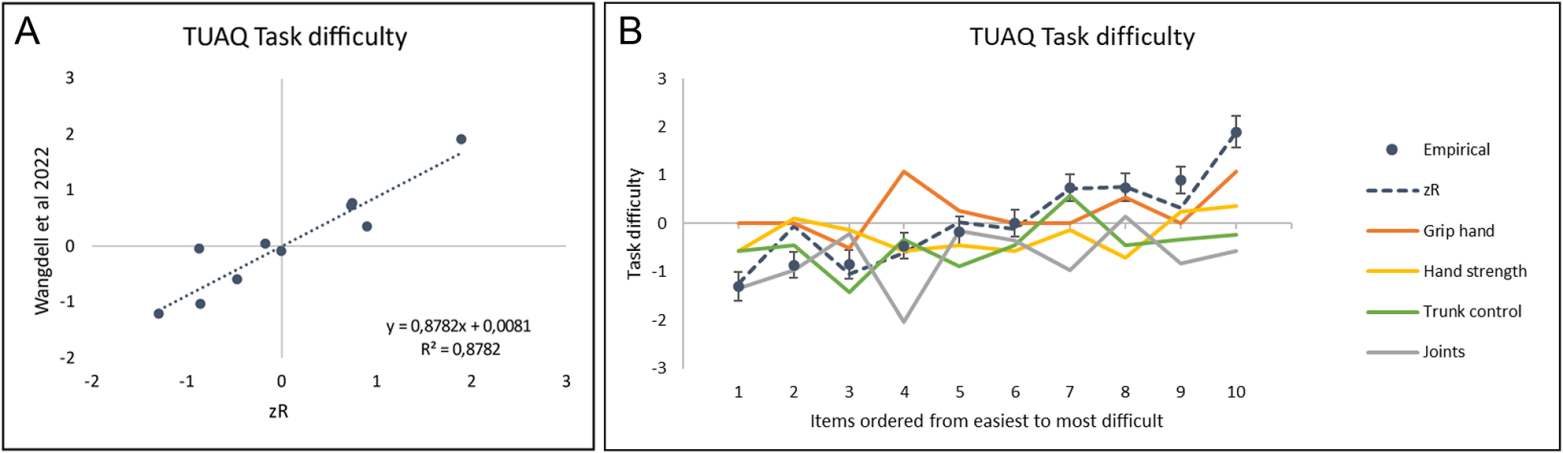
(**A**) Linear regression of the observed TUAQ task difficulty, *δ*, from Wangdell et al.^7^ against the CSE predicted zR for the TUAQ. (**B**) Predicted contributions, $\Delta$ *δ*, to task difficulty from the four explanatory variables from Eq. 3. Items are ordered from the easiest to the most difficult according to Wangdell et al.^7^ as also reported in Table 2.

That is, of the initial ten candidate explanatory variables for task difficulty, only four were chosen on the basis of their degree of univariate correlation, namely *Hand grip*,* Hand strength*,* Trunk control and Joints* (Table 4). Figure 1A shows the correlation plot of the estimated UL task difficulty from the CSE *zR*with the UL task difficulty, , from Wangdell et al. 2022^8^, resulting in a Pearson correlation coefficient of 0.94. This CSE was the most promising of the different versions tested in terms of the best correlation coefficient and the lowest measurement uncertainties in the $\beta$-coefficients. Figure 1B shows the contribution of each explanatory variable to each TUAQ item. Contribution refers to “how much” each explanatory variable helps explain the difficulty of individual items on the TUAQ scale. A positive coefficient indicates a positive contribution, that is, when the explanatory variable increases the difficulty of an item also increases, and vice versa. More specifically, the coloured lines in Fig. 1B provide a visual representation of the contribution of each explanatory variable to each item.

### A construct specification equation (CSE) for person UL ability, 

A corresponding correlation matrix for person UL ability, , and potential explanatory variables presented in Sect."Tetraplegia Upper Limb Activities Questionnaire (TUAQ)"is presented in Table 5. By including all potential explanatory variables, it yielded the following CSE:

3 $$\begin {aligned} {zR}_{TUAQPerson\,ability} &=-3\left(1\right)+0.09\left(6\right)\cdot{{\theta}_{TPI}}_{i}+0.07\left(8\right)\cdot{{\theta}_{TPPS}}_{i}+0.8\left(7\right)\cdot{{\theta}_{pU{L}{surg}}}_{i}\\&+0.05\left(7\right)\cdot{{\theta}_{Grip\,strength}}_{i}-0.08\left(22\right)\cdot{{\theta}_{Pinch\,strength}}_{i}+0.019\left(9\right)\cdot{{\theta}_{GRT}}_{i} \end {aligned}$$

**Fig. 2:**
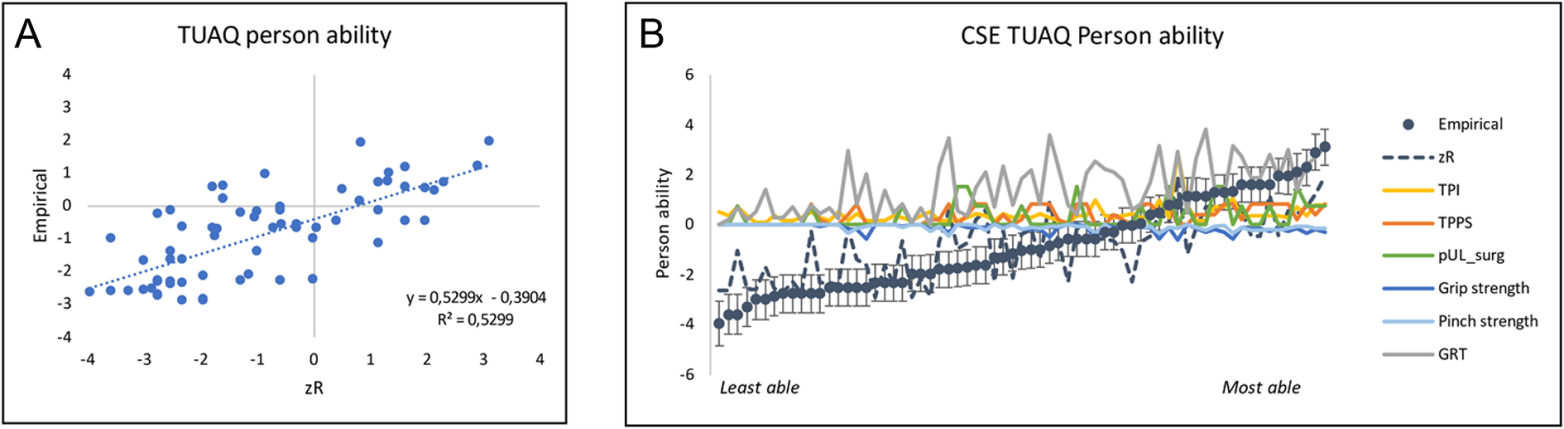
(**A**) Linear regression of the observed TUAQ person ability, θ, against the CSE predicted zR for the TUAQ. (**B**) Predicted contributions,$\Delta\vartheta$, to person ability from the six explanatory variables from Eq. (4) GRT = Grasp and release test; pUL_surg = Previous UL surgeries; TPI = Time post injury; TPPS = Time pre or post-surgery.

The Pearson correlation coefficient was 0.73 between estimated person UL from the CSE *zR* with person UL ability, (Fig. 2A). Despite rather high univariate correlations (Table 5), grip- and pinch strength show larger measurement uncertainties than their β-coefficients. Likewise, TPPS demonstrated large measurement uncertainties with a weaker univariate correlation. Consequently, removing these variables did not significantly improve the predictive power. The little contribution from those variables is shown in Fig. 2B, where the lines are close to zero with little variation along the ability continuum. Furthermore, GRT seems to be the dominating explanatory variable for person UL ability, .

**Table 5:** Pearson correlation coefficients between estimates of person ability against all six potential explanatory variables, $\theta_{x_{i}}$ (section"Tetraplegia Upper Limb Activities Questionnaire (TUAQ)").

	Person hand ability	TPI	TPPS	pUL_surg	Grip strength	Pinch strength	GRT
TPI,${{\theta}_{TPI}}_{i}$	0.18						
TPPS,${{\theta}_{TPPS}}_{i}$	0.09	−0.03					
pUL_surg,${{\theta}_{pU{L}_{surg}}}_{i}$	0.21	0.55	0.09				
Grip strength,${{\theta}_{Grip\,strength}}_{i}$	0.41	0.55	0.25	−0.03			
Pinch strength,${{\theta}_{Pinch\,strength}}_{i}$	0.39	0.24	0.44	−0.10	0.63		
GRT,${{\theta}_{GRT}}_{i}$	0.39	0.20	0.40	−0.12	0.72	0.66	

GRT = Grasp and release test; pUL_surg = Previous UL surgeries; TPI = Time post injury; TPPS = Time pre or post-surgery.

## Discussion

To our best knowledge, this is one of the first papers to provide simultaneously two CSEs: one for UL task difficulty, δ, and one for person UL ability, . A good CSE for task difficulty, , supports the construct validity of the TUAQ when measuring person UL ability, and the CSE for person ability, , can also be used both for validation as well as a clinical tool. The CSE for task difficulty provides primary validity for the measured construct task difficulty, *δ*, while the CSE for person ability provides primary validity for the measurement of person ability, . With the results of this paper, we further strengthen the validity and the understanding of both conjoint attributes in themselves as well as the attributes as measured.

A Pearson correlation coefficient of 0.94 between the predicted *zR* and the measured UL task difficulties, *δ*, is very strong. There are, however, two things challenging our results:

First, as shown in Fig. 1B, item 4 – *Shaving/put on makeup* –deviates somewhat from our overall predictions. It might be related to the type of item, as this is a personal choice and therefore not done by everyone on a daily basis. There is also an overall trend across the items where the “Grip hand” curve is mostly positive and lies above the “Joints” curve, which is mostly negative. The difference in sign of these two contributions to task difficulty agrees with the opposite signs (last two terms of the CSE, Eq. 3) to the overall task difficulty. What is striking in Fig. 1B is in item 4 where there are relatively large deviations for the explanatory variables *Demand for hand grip* and *Joints* (Table 3). Uncertainties are admittedly large. But one explanation could be that as an individual item this task has the highest values of both *Demand for hand grip* and *Joints*, while most other tasks with high *Demand for hand grip* have less engagements from *Joints*, and vice versa. The degree of motoric precision required for this task e.g., not accidently cutting oneself when using a razor compared to a task such as handling money where injury is less likely with imprecision may explain this. Another explanation might be that the task comprises two related but also somewhat different tasks making rating difficult.

Secondly, the absence of correlation between the measured task difficulty, *δ*, and the explanatory variable *Sequence complexity*contradicts our initial hypothesis that the item hierarchy indicated that easier items were less complex (e.g., fewer joints involved and less coordination between left and right arms) compared to the more difficult items^8^. One possible explanation for this could be that especially individuals with tetraplegia might use aids or adjust to their environment for the easier tasks compared to the more challenging tasks, and therefore, were rated as easier in the initial study.

These two aspects – deviations for item 4 and for *Sequence complexity*, together with other advancements in the field – including our own work^12,23^– about the role of entropy to explain task difficulty, warrant further investigation. Entropy as an explanatory variable for both task difficulty and person ability should reasonably apply also to other kinds of human abilities and tasks in health care. Translating our previous findings into hand tasks studied here implies that we would expect there to be more order (less entropy) in the easiest item – *Eating with a fork or spoon* – compared with a medium difficult item – *Pick up items from a flat surface* – and compared with the most difficult item – *Cut food when eating*. The same concept has indeed been used previously by Iosa et al.^30^ who used informational entropy to estimate the complexity of a hand trajectory, and hence relating it inversely to the efficacy of hand movements performed to complete a task. This is the topic of future studies.

When testing this in forthcoming work, it will be important to remember that the focus when seeking to explain task difficulty assumes that there should be some general demands on the body to perform different tasks, which is different from explaining an individual person’s ability^23^. As proposed elsewhere, when extending CSE beyond immediate quantitative explanatory variables (such as sequence length, word frequency)^12,15–18^, one may use a group of people to define the explanatory variables^23^. It is likely that a group of people who can all perform all tasks equally well will have a very low variation in an entropy measure of, for instance, the complexity of the trajectory and the average entropy is expected to increase linearly with the difficult of the tasks^23^. Conversely, in a group of people with varying ability it is likely that each person’s entropy measure for a single hand task will also vary, which could be used to explain his or her UL ability. In this paper, we have not included such variables, but have instead chosen typical person characteristics collected in the clinic.

The aim of the TUAQ is to capture daily tasks, independent of technique^8^, rather than studying specific functions, such as grip ability or strength. The CSE complements the measurement of a person UL ability with specific functions required to generally perform tasks along the continuum. This can guide clinical decisions and target interventions such as training at the functional level, use of specific aids or compensatory techniques^31,32^. This study showed somewhat improved univariate correlations between person UL ability, and GRT and Pinch, respectively, compared to a previous study^9^. Similar to the previous study, the sample size is small, and the measurement uncertainties are too large to make any finite conclusions about the differences beyond sampling issues or measurement noise. The Pearson correlation coefficient of 0.73 between the estimated person UL ability from zR from the CSE and the measured person UL ability, , however gives support for seeking multivariate explanations as opposed to univariate explanation. At the same time, there are possible additional explanatory variables not yet included in the CSE that may further improve the understanding of what is causing variation in person hand ability. In Fig. 2A it is evident that there are individuals who have a better measured UL ability than predicted (left top of the regression line) and individuals who have a worse UL ability than predicted (left bottom of the regression line). Here, we may speculate that a better UL ability than predicted may be explained by person characteristics related to creativity and motivation to learn new skills while a worse UL ability than predicted may be explained by an unsuccessful surgery or secondary complications, which is not captured in the CSE. In addition, on the upper end of the continuum there is less scatter, and it seems to be easier to explain the variation in UL ability. One possible reason for this could be a sampling issue. Thus, future work would benefit from including a larger sample that would allow exploration of variations due to injury level, and incomplete versus complete injury. Furthermore, the present CSE is dominated by GRT followed by grip strength, both of which specifically address grasp and not variables related to the full UL. We therefore recommend further work to investigate the role of variables such as elbow and/or shoulder function, compensatory strategies, sensation and neuropathic pain when explaining person UL ability.

While this study has strength in terms of exploring and extending methodology into new areas, it has its limitations. Despite the good prediction for UL task difficulty, some explanatory variables (such as complexity) did not follow our theory, thus warranting further investigation. Furthermore, we had some difficulty in recruiting healthcare professionals to provide the ratings. However, we successfully engaged experts from the largest and most prominent clinics worldwide. While we can conclude that the study worked well for those who responded, further research is needed to recruit more respondents to provide stability in explanatory variables. This work should include evaluations of stability in the estimates of explanatory variables across professional, working experience and education. Further work also needs to investigate appropriate sample size when using professionals to rate explanatory variables. Moreover, we would suggest that alternative explanatory variables – such as dividing hand grip, hand strength and arm strength for dominant and non-dominant hand into separate explanatory variables as well as entropy measures of trajectory complexity – should be investigated. With respect to explaining person UL ability, we used existing data rather than collecting new data for this study. We believe that this is a feasible approach, but as a next step, more well-designed data collection of explanatory variables for person UL ability (e.g., trunk stability and shoulder engagement) will advance the results further.

Recently, McKenna and colleagues^33^ argued that there are no patient-reported outcome measures with coherent construct definitions, satisfying fundamental and unidimensional measurement properties, and CSE, leading them to question existing practice. We agree that all three components are essential, but to reach such practice, methodological work is warranted to (i) understand and agree upon the best practice for CSE and (ii) test, evaluate, and show the applicability of CSEs across many more constructs. In the present study, we started with a conventional Rasch analysis to derive separate measures of UL task difficulty and UL person ability followed by a PCR for formulation of the CSEs in support of the validity of these two constructs. The PCR procedure has the advantage of taking into account all explanatory variables which may not be experimentally observed quantities, by “rotation” in the explanatory-variable space from the experimental dimensions to the principal component dimensions^12,23^. A detailed comparison of the PCR method used here with other methods, such as exploratory IRT models (e.g., linear logistic test model (LLTM)) or regression models (e.g., partial least squares (PLS) or lasso), is recommended. Such a comparison should include both the investigation of contributions from explanatory variables and predictive power as well as conceptual justifications, consequently, it will provide best practice for CSE. The “goodness of fit” of each CSE model to the observed task difficulties and person abilities, perhaps limited by sample size as well as other factors, can be judged in a number of ways. As found sufficient in the original CSE work of Stenner et al.^15,16^, one approach adopted – common to both the present PCR and simpler regressions, such as the linear - is to examine the uncertainties in each coefficient shown in the parentheses in Eqs. 2 and 3, respectively, as said already at the lines after Eq. 2, together with evaluations of Pearson correlation coefficients in each regression. As explained in our earlier publications^12^, these uncertainties include in general contributions from various factors, such as uncertainties in the input data and uncertainties when making a regression of the model to the data. There are of course other goodness-of-statistics, beyond that considered sufficient^15,16^ which might have been chosen, such as the popular Information Criteria AIC and BIC in making inferences on models and PCA loading studies^34^ which might be investigated further. In the current work, we have made a pragmatic choice which aims to minimise overburdening the practitioner with elaborate statistics.

In addition to the new findings related to UL measurements for people with tetraplegia and support of the validity of TUAQ, these results also contribute with evidence of the applicability of CSEs for another construct. Nevertheless, most CSEs have been attached to unique scales, such as EDAQ^20^, the Gross Motor Function Measure-66 (GMFM)^21^, gross upper-extremity function (GUE)^36^, and TUAQ in the present study. Nevertheless, CSE also ‘*provides a means to calibrate most*,* if not all*,* items measuring a construct onto the same scale’*to provide universal measurement units^21^. Examples of this are given in our previous work, where we have demonstrated how to unify a single unidimensional construct for short-term memory based on both forward and backward sequences and visuospatial and verbal memory tasks^37^.

To conclude, while much of the work on CSE in other fields (such as memory) has focused on task difficulty, , and used quantitative explanatory variables, the present work has both extended and explored the methodology for using more qualitative explanatory variables. Specifically, for UL measurements for people with tetraplegia, a good CSE for task difficulty, , was formulated – using qualitative explanatory variables – in support of the validity of TUAQ when measuring person UL ability. The CSE formulated for person ability, , can be used both for validation purposes and as a clinical tool. While the conjoint scale for person ability and task difficulty provides clinicians with insights into the types of tasks a person with a specific ability level is likely to perform, the CSE for UL person ability offers a deeper understanding of the factors contributing to variations in UL abilities across the continuum. By utilizing the CSE for UL person ability, clinicians gain more detailed information on the underlying factors that influence a patient’s ability, enabling them to target specific areas in rehabilitation to enhance the patient’s ability. The presented CSE development for UL task difficulty and person ability warrants further development but already provides promising evidence to be used both for validation and as a clinical tool.

## Electronic supplementary material

Below is the link to the electronic supplementary material.

Supplementary Material 1

## Data Availability

The data that support the findings of this study are not openly available due to reasons of sensitivity and are available from the corresponding author upon reasonable request.
