# Supplementary material for: An investigation of improving validity in upper limb measurements for people with tetraplegia using construct specification equations
Source: Sci Rep. 2025 Aug 4;15:28468. doi: 10.1038/s41598-025-98626-4 (PMC12322045; doi:10.1038/s41598-025-98626-4)
Supplement: Supplementary file 1 — Supplementary Material 1 [file 41598_2025_98626_MOESM1_ESM.pdf]

*Supplementary Material*

# An Investigation of Improving Validity in Upper Limb Measurements for People with Tetraplegia Using Construct Specification Equations

**Johanna Wangdell[1, 2 *], Leslie Pendrill[3], Jennifer A. Dunn[4], Bridget Hill[5, 6], Jeanette Melin[3, 7]**

*** Correspondence:** Corresponding Author: johanna.wangdell@vgregion.se

Supplementary 1. TUAQ items ordered from easiest to most difficult according to Wangdell et al (7) and measurement values for each explanatory variable, .

| | TUAQ items / Explanatory variables | Hand grip | Hand strength | Arm strength | Stabiliz-ation position-ing arm | ROM Domi-nant hand | ROM Not Domi-nant hand | Trunk control | Coordi-nation | Seque-nce complexi ty | Joints |
|---|---|---|---|---|---|---|---|---|---|---|---|
| 1 | Eating with a fork or spoon | 0.01 (1.26) | -3.45 (1.74) | 1.89 (1.32) | 1.68 (1.58) | -0.47 (1.24) | -0.9 (1.24) | -1.38 (1.12) | 1.24 (1.84) | -0.5 (1.4) | 2.73 (1.38) |
| 2 | Drinking from a bottle | 0.01 (1.26) | 0.69 (1.82) | 2.32 (1.3) | 1.08 (1.54) | -0.08 (1.26) | -1.16 (1.24) | -1.08 (1.08) | 0.48 (1.72) | -2.65 (1.62) | 1.96 (1.14) |
| 3 | Grasp and reposition book/tablet | -0.78 (1.26) | -0.82 (1.66) | 0.51 (1.44) | -1.6 (1.5) | -1.25 (1.28) | -1.16 (1.26) | -3.46 (2.1) | -0.31 (1.86) | -1.5 (1.44) | 0.43 (0.96) |
| 4 | Shaving/put on makeup | 1.66 (1.32) | -3.45 (1.74) | 2.32 (1.3) | 1.68 (1.58) | 0.32 (1.28) | 0.3 (1.22) | -0.8 (1.04) | 3.16 (3.72) | 4.22 (1.92) | 4.14 (2.16) |
| 5 | Writing with a pen (with your best hand) | 0.41 (1.28) | -2.74 (1.62) | -1.38 (1.68) | -1.06 (1.46) | -0.85 (1.24) | -2.72 (1.66) | -2.13 (1.34) | 0.96 (1.72) | 0.51 (1.46) | 0.3 (0.9) |
| 6 | Pick up items form a flat surface | 0.01 (1.26) | -3.45 (1.74) | -0.05 (1.54) | -2.21 (1.6) | -1.67 (1.32) | -2.02 (1.44) | -1.08 (1.08) | 0.27 (1.86) | -2.65 (1.62) | 0.71 (0.92) |
| 7 | Adjust upper half clothing pulling down back to waist level | 0.01 (1.26) | -0.82 (1.66) | 3.18 (1.32) | 1.08 (1.54) | -0.08 (1.26) | -0.41 (1.20) | 1.4 (1.16) | -0.12 (1.72) | 0 (1.42) | 1.96 (1.14) |
| 8 | Handle banknotes credit card in/out of wallet | 0.82 (1.28) | -4.31 (2) | 1 (1.38) | -1.6 (1.5) | -1.25 (1.28) | -1.43 (1.26) | -1.08 (1.08) | 0.96 (1.72) | 0.51 (1.46) | -0.28 (0.88) |
| 9 | Open previously opened jars | 0.01 (1.26) | 1.5 (1.78) | 1.45 (1.34) | -0.02 (1.44) | 0.32 (1.28) | 0.54 (1.18) | -0.8 (1.04) | -0.49 (1.86) | -2.04 (1.5) | 1.66 (1.06) |
| 10 | Cut food when eating (with your best hand) | 1.66 (1.32) | 2.25 (1.7) | 2.75 (1.32) | 1.68 (1.58) | -1.25 (1.28) | -0.65 (1.24) | -0.54 (1.02) | 1.24 (1.84) | -0.99 (1.4) | 1.15 (0.96) |
| | Reliability | 0.66 | 0.94 | 0.45 | 0.48 | 0.47 | 0.53 | 0.48 | 0.24 | 0.50 | 0.68= |
